# Prevalence, estimated HIV-1 incidence and viral diversity among people seeking voluntary counseling and testing services in Rio de Janeiro, Brazil

**DOI:** 10.1186/1471-2334-10-224

**Published:** 2010-07-28

**Authors:** Carlos A Velasco de Castro, Beatriz Grinsztejn, Valdiléa G Veloso, Francisco I Bastos, José H Pilotto, Mariza G Morgado

**Affiliations:** 1 Laboratório de AIDS & Imunologia Molecular, Instituto Oswaldo Cruz, Fundação Oswaldo Cruz, Rio de Janeiro, Brasil; 2 Instituto de Pesquisa Clínica Evandro Chagas, Fundação Oswaldo Cruz, Rio de Janeiro, Brasil; 3 Instituto de Comunicação e Informação Científica e Tecnológica em Saúde, Fundação Oswaldo Cruz, Rio de Janeiro, Brasil; 4 Hospital Geral de Nova Iguaçu, Nova Iguaçu, Rio de Janeiro, Brasil; 5 Laboratório de Virologia, Departamento de Patologia Clínica, Instituto Fernandes Figueira, Fundação Oswaldo Cruz, Rio de Janeiro, Brasil

## Abstract

**Background:**

BED-EIA HIV-1 Incidence Test (BED-CEIA) has been described as a tool to discriminate recent (RS) from long-term (LTS) seroconversion of HIV-1 infection, contributing to a better understanding of the dynamics of the HIV/AIDS epidemic over time. This study determined the prevalence, estimated incidence and HIV-1 subtype infection among individuals seeking testing in Voluntary Counseling and Testing centers (VCTs) from Rio de Janeiro, Brazil.

**Methods:**

Demographics and behavioral data were obtained from 434 individuals, diagnosed as HIV-positive among 9,008 volunteers screened from November 2004 to October 2005 in three VCTs located in the Rio de Janeiro Metropolitan area, Brazil. BED-CEIA protocol was performed to identify RS. DNA samples from RS and a subset of LTS (under a proportion of 1:2) were selected for gp120 C2-V3 and *pol *(protease and reverse transcriptase) regions genomic sequencing.

**Results:**

Overall HIV-1 prevalence was 4.8%. Sixty-one of 434 seropositive individuals were classified as RS, corresponding to an incidence rate of 1.68%/year (95%CI 1.26% -2.10%). Estimated incidence between Men Who Have Sex with Men (MSM) was 11 times higher than among heterosexual men and 55% of the new cases were identified in volunteers aged 25-40 years. A similar distribution of different HIV-1 subtypes was found among RS and LTS.

**Conclusions:**

Our data suggest that prevention for MSM remains a challenge and efforts focusing on prevention targeting this population should be prioritized. No significant changes in HIV-1 subtypes were observed among the RS and LTS subgroups. One case of HIV-1 AUK (pol)/A (env) recombinant genome was detected for the first time in Brazil.

## Background

The HIV pandemic continues to spread unabated, with about 33.4 million people living with HIV worldwide. Globally, approximately 2.7 million new infections are believed to occur in 2008 [1]. Hosting the largest HIV-1 infected population in Latin America, Brazil has a complex HIV-1 epidemic characterized by an increase of women participation over time, a pronounced decrease among injecting drug users and persisting high-risk behaviors and a higher proportion of new infections among gay and bisexual men [2]. Approximately 620,000 individuals aged 15 to 49 years old are estimated to be infected with HIV in Brazil. According to the former National AIDS Program (recently renamed as the Department of STD, AIDS and Viral hepatitis) 544,846 AIDS cases were reported from 1980 to June 2009. Rio de Janeiro is the second Brazilian state in terms of number of reported AIDS cases, corresponding to 14% of the overall national AIDS cases [3]. Sentinel studies in pregnant women estimate an overall prevalence of HIV-1 infection of 0.61% [4]. Serology for HIV infection is available at no cost in the Voluntary Counseling and Testing centers VCTs) all over the country, where the volunteers have full access to prevention activities and individual counseling.

In Brazil, twenty years ago, facilities offering voluntary counseling and testing (VCT) at not cost at the point of delivery were established as a key strategy to promote the access of the Brazilian population to early diagnosis and prevention of HIV and others sexually transmitted diseases (STDs) and to stimulate agile referral to treatment. Such VCT centers became a central piece of Brazil's initiative to curb the epidemic over the years and nowadays comprise a comprehensive network of over 380 public health facilities spread all over the country. In cities with VCT centers, the number of HIV tests per 1,000 people is 2.4 times higher than in the localities where there is no VCT center. At present, in Rio de Janeiro state, 13 VCT centers are fully operative - eight of them located in the metropolitan area [5].

In order to better understand recent epidemiological trends in HIV transmission it is key to estimate incidence and to assess viral diversity among recently infected individuals. Serological differentiation between recent (RS) and long-term seroconverters (LTS) has been described as a powerful tool to track the trends of the HIV/AIDS epidemic [6]. A variety of approaches have been described over time [7,8]. One of those assays is based on the capture and detection of increasing proportions of HIV-1 IgG in the serum over the time (BED-CEIA) that uses a branched peptide based on gp41 of B, D and E HIV-1 subtypes [9,10]. Due to some concerns that have emerged in scenarios with high HIV prevalence and/or viral diversity [11], two correction strategies/factors were proposed to minimize the putative overestimation of incidence rates using BED-CEIA. One of them proposes the exclusion of certain specimens on clinical grounds, by relying on trend differences rather than absolute incidence estimates, using secondary confirmatory testing or adjustments for misclassification [12]. The second correction factor adjusts the findings in order to avoid misclassification of subtype C infected individuals [13].

Brazil is a huge country presenting heterogeneous HIV/AIDS epidemiological trends [3] and virological scenarios, with distinct HIV-1 subtypes and recombinant infections [14] in different regions and/or population strata. The aim of this study was to assess the prevalence and to estimate incidence of HIV-1 infection among individuals seeking HIV testing in VCT centers located in the Rio de Janeiro Metropolitan area, as well as to track HIV diversity among RS and LTS over time.

## Methods

### Study Population

Frozen sera collected from a consecutive sample of individuals from November 2004 to October 2005 (n = 9,008), recruited from three VCT centers located in municipalities of Rio de Janeiro Metropolitan area were analyzed in this study. The VCT centers were selected based on their role as reference centers for people seeking HIV testing at no cost in the Rio de Janeiro Metropolitan area. These sites are the places where any Brazilian citizen can be tested for HIV infection for free, keeping anonymity if desired, with easy access, and in the context of a welcoming environment. They are localized mainly in the outskirts of Rio de Janeiro, which corresponds to a catchments area of about 5 million people, the vast majority of them from poor social strata.

Demographic information was obtained from baseline data collection forms, administered in private by trained interviewers. Among the population studied, the records provided information on gender, age and sexual orientation for all individuals. In 96.5% of HIV-1 seroreactive individuals (419 of 434) it was possible to obtain additional information on testing such as: reasons for being tested, previous testing, and presence of symptoms associated to HIV/AIDS, as well as a history of risky injection behaviors. We should observe here that the protocol of the present study did not interfere in any sense with the routine procedures adopted by each one of the VCT centers and in this sense allows for individuals the right to refuse to answer one or more questions they may consider as embarrassing or annoying. Individuals were entitled to be counseled and tested, as defined by the guidelines of the Brazilian Ministry of Health (BMoH), irrespectively of their answers (or the absence of answers) to the short standard form used as a routine procedure in the VCT centers.

The present research protocol conformed to the Helsinki Declaration and to the local legislation. Informed consent was obtained from the participants and the refusal to provide information perceived as sensitive explicitly guaranteed by the study protocol. The study was approved by the Oswaldo Cruz Foundation (FIOCRUZ) - Evandro Chagas Clinical Research Institute Ethical Board, registration CAAE-0032.0.009.000-04.

### Serological testing algorithm

With the goal of enabling this research protocol without disturbing the routine procedures of the VCT centers, two parallel blood tubes (BD Vacuntainer^® ^ref. 367958) were collected from each individual. One of them remained in the VCT centers, in order to conduct the standard serological testing and delivery of results with the corresponding post-test counseling, in accordance with the Brazilian Ministry of Health (Ordinance N°59 of January 28, 2003). The second tube was sent to the Laboratory of AIDS & Molecular Immunology, IOC (FIOCRUZ), and then stored at -20°C until the delivery of the results of the HIV testing. This strategy assured the non-interference in the VCT center routine, as well as the possibility to have 100% of the HIV seroreactive samples viable to perform BED-CEIA methodology. These samples were then thawed, aliquoted into sterile microtubes and stored at -70°C for subsequent studies. Two aliquots of serum and one aliquot containing the blood clot were available for each HIV seroreactive sample.

In order to identify recent HIV-1 infections, serum samples reactive for HIV antibodies were subjected to testing in vitro by a quantitative competitive capture enzyme immunoassay (BED-CEIA), to determine the proportion of anti-HIV-1 specific IgG in relation to the total IgG, according to the manufacturer's instructions [9]. Briefly, serum (or plasma) samples were added to the wells of a microplate coated with anti-human IgG. HIV specific and not specific IgG molecules were captured on the solid phase, representing populations of antibodies found in the sample. After the incubation and washing protocol, a synthetic chimeric polypeptide containing the HIV-1 immunodominant epitope of gp41 subtype B, E and D was added and incubated for one hour at 37°C. At this stage the peptide bound to their respective antibodies (anti-HIV-1 specific). After further steps the reactions is revealed in the presence of TMB (tetramethylbenzidine) and read using a spectrophotometer (wavelength 450 nm). The color intensity obtained in each sample is directly proportional to the amount of specific anti-HIV-1 antibodies. The controls (negative, low positive and high positive) (Calypte HIV-1 BED Incidence EIA) and calibrators are analyzed along with samples. The criteria for validation of the test, definition of samples to confirm and set the time of infection were used in accordance with the instructions and worksheet provided by the manufacturer [10]. Annual HIV seroincidence and 95% confidence intervals (CIs) were calculated on the basis of the BED-CEIA results, using the 153-day window period and the seroincidence formula described elsewhere [9,10]. Two correction strategies, here named CF1 [12] and CF2 [13] were further applied.

### DNA Extraction and PCR amplification of HIV-1 env and pol regions

Proviral DNA was extracted from the blood clot using the phenol/chloroform method [14]. The samples were amplified for the gp120 C2-V3 region using a nested PCR protocol with ED5/ED12 and ED31/ED33 oligonucleotide sets respectively as outer and inner primers [15], generating a fragment of approximately 564 bp. Similarly, for the *pol *gene we amplified a 1 Kb fragment covering the protease (PR) and part of the reverse transcriptase (RT) using the outer DP10/LR54 and inner DP16/LR49 primer sets as previously described [16,17]. The samples were amplified in the thermocycler GeneAmp^® ^PCR System 9700 (Perkin Elmer - Norwalk, USA) using the following conditions: 3 cycles at 97°C for 1 minute, 55°C for 1 min, 72°C for 2 minutes. Thirty two cycles at 94°C for 45 seg, 55°C for 1 minute, 72°C for 2 minutes. Final cycle was performed at 72°C for 10 minutes. PCR products were visualized after electrophoresis in 1% agarose gels (Gibco/BRL, Life Technologies - USA) and purified (GFX™ PCR DNA and Gel Band Purification Kit) following the manufacturer's protocol (Amersham Biosciences, GE Healthcare - UK).

### DNA sequencing and Phylogenetic analyses

Purified PCR products corresponding to *env *and *pol *regions were respectively submitted to automated sequencing using the Big Dye Terminator Cycle Sequencing Ready Reaction - ABI PRISM (Perkin Elmer, Foster City, USA), according to manufacturer's instructions. Fifty to 100 ng of amplified DNA were used for sequencing reaction in both senses using the *env *inner primers ED31 and ED33. For the *pol *region, sequence reactions were performed using DP16 and LR49 in addition to the RT12 and LR51 in order to cover the whole fragment in both directions Sequencing reactions were performed in the thermocycler GeneAmp^® ^PCR System 9700 (Perkin Elmer - Norwalk, USA) using the following conditions: 25 cycles of 96°C for 30 seconds, 50°C for 20 seconds and 60°C for 4 minutes. Each reaction product with a final volume of 20 μl was incubated for 15 min with 80 μl of 75% isopropanol, followed by centrifugation, discarding of the supernatant and drying the precipitate in the thermalcycler at 70°C for about 10 minutes. The pellet was suspended with 10 ul of Hi-Di Formamide, followed by a shock of 5 minutes at 95°C and 1 minute on ice for denaturation and placed on the ABI model 3100 DNA Sequencer (Applied Biosystems, Foster City, CA).

Generated DNA sequences (sense and anti-sense) of each sample were assembled using SeqMan, included in the DNASTAR package [18]. Sequence electropherograms obtained for the *env *C2-V3 and *pol *regions were respectively aligned against a set of reference strains from HIV-1 group M subtypes using CLUSTAL X [19]. SIVcpz *env *C2V3 or *pol *sequences were used as outgroup in both analyses. Gap stripping and minor adjustments were manually performed. Phylogenetic inferences were performed by the neighbor-joining algorithm using the program Mega 3.0 (Molecular Evolutionary Genetics Analysis, version 3.0), and the reliability of the branches determined by analysis of bootstrap calculated based on 100 re-samplings [20]. TREEVIEW [21] was used for visualization of the phylogenetic trees. Screening of *pol *recombinant forms were assessed through the Rega HIV-1 Subtyping Tool website [22] and further confirmed by bootscanning analysis (sliding window of 400 bp, incremental steps of 10 bases, and the Kimura two-parameter model) as implemented in Simplot version 2 (Ray S. Simplot v2.5.0 http://sray.med.som.jhmi.edu/SCRoftware/simplot/).

### Data Analysis

Data were entered into contingency tables and analyzed using chi-square or Fisher's exact test.

## Results

Overall 9,008 persons were tested for HIV infection in the three VCT centers. Men comprised 41.4% of the tested population, of whom 10.7% (n = 399) referred to have had sex with other men. Twenty-eight percent of clients were younger than 25 years, 40.8% were 25-40 years old, and 31.2% were older than 40 years.

The overall prevalence of HIV infection was 4.8%, higher for men compared to women (6.5% *vs*. 3.6%). Prevalence was 6 times higher for MSM (24.8%) vis-à-vis heterosexual men (4.3%). The age group more affected was young adults aged 25 to 40 years old, with a prevalence of 6.3% (Table 1). The major motivations for testing could be obtained for the HIV seropositive individuals. In general, about thirty-five percent of the individuals with valid answers admitted to be recently exposed to risk as the main reason to seek and HIV test, over one quarter said testing had been taken after the suggestion made by professionals from a health service. Forty individuals (roughly corresponding to 10% of the individuals with valid answers) reported that symptoms related to HIV/AIDS were their main motivation for testing, whereas 15% reported they were mainly motivated by the desire to prevent themselves against HIV. In this subgroup, only five people were returning for a new test (all of them with prior negative results), and 404 individuals with valid answers (96.42%) reported unprotected sexual intercourse as their main risk factor. Eight individuals (less than 2%) reported to have shared syringes in the context of illicit substance self-administration.

**Table 1:** Prevalence of HIV-1 infection in 9,008 individuals screened in 3 VCTs of Rio de Janeiro state from November 2004 to October 2005.

Variables (n)	Seropositives		P value
	%Prevalence (n)	95%Confidence Interval	
**Overall**= 9,008	4.8 (434)	4.4 - 5.3	-
**Gender**			
Female (5,282)	3.6 (191)	3.1 - 4.1	***< 0,0001***^***a***^
Male (3,726)	6.5 (243)	5.7 - 7.3	
**Sexual practice (men)**			
Heterosexual (3,327)	4.3 (144)	3.6 - 5.0	***< 0,0001***^***a***^
MSM (399)	24.8 (99)	19.9 - 29.7	
**Age - years**			
< 25 (2,525)	2.6 (65)	1.9 - 3.2	***< 0,0001***^***b***^
25 a 40 (3,671)	6.4 (233)	5.5 - 7.1	
> 40 (2,812)	4.8 (136)	4.0 - 5.6	

a = *X*^2^

b = Fisher's exact test

Based on the BED serology results, sixty-one out of the 434 seropositive individuals (14.0%) were classified as RS, with an overall incidence rate of 1.68%/year (95%CI 1.26% -2.10%). Data including the estimated incidence re-calculated according to CF1 (1.09%/year) and CF2 (1.17%/year) are also presented. Incidence rates were also calculated according to the different exposure categories and age strata (Table 2). Overall, higher incidence rates were observed for MSM when compared to heterosexual men (8.55-11.96% *vs *0.56-1.12%), and young adults aged 25-40 years old.

**Table 2:** Estimated HIV-1 Incidence of HIV infection among 9,008 individuals (434 seropositive samples) screened in 3 VCTs of Rio de Janeiro Metropolitan area from November 2004 to October 2005 - conventional estimation and estimation with 2 corrective factors (CF).

	Recent Infections	**Estimate Incidence**^**a**^ %/year (95% CI)	**Estimate Incidence**^**b**^ %/year (95% CI)	**Estimate Incidence**^**c**^ %/year (95% CI)
**Overall = 434**	61	1.68 (1.26 - 2.10)	1.09 (1.32 - 1.87)	1.17 (0.88 - 1.47)
**Gender**				
Female (191)	30	1.40 (0.90 - 1.90)	0.97 (0.62 - 1.31)	1.04 (0.67 - 1.41)
Male (243)	31	2.10 (1.36 - 2.84)	1.27 (0.82 - 1.72)	1.37 (0.89 - 1.85)
**Exposure categories** **(among men)**				
Heterosexual (144)	15	1.12 (0.55 - 1.68)	0.56 (0.28 - 0.84)	0.60 (0.30 - 0.91)
MSM (99)	16	11.96 (6.10 - 17.82)	8.55 (4.36 - 12.74)	9.17 (4.68 - 13.67)
**Age (years)**				
<25 (65)	9	0.87 (0.30 - 1.44)	0.56 (0.19 - 0.92)	0.60 (0.21 - 0.99)
25 to 40 (233)	34	2.33 (1.55 - 3.12)	1.55 (1.03 - 2.07)	1.67 (1.11 - 2.23)
>40 (136)	18	1.59 (0.86 - 2.33)	0.99 (0.53 - 1.45)	1.07 (0.57 - 1.56)

^a ^Incidence estimated with conventional estimation [9]

^b ^CF1 = Incidence estimated with Corrective Factor 1 [12]

^c ^CF2 = Incidence estimated with Corrective Factor 2 [13]

Molecular analyses of the C2-V3 *env*, RT and Protease genomic regions were performed for 52 out of the 61 RS (85%) and 114 out of the 122 (93%) randomly selected LTS samples. Overall, 80.1% (n = 133) individuals were infected by subtype B viruses, 8.4% by subtypes F (n = 13) and C (n = 1), and recombinant forms were found in 11.4% (n = 19) of the isolates. No significant differences were found between RS and LTS for subtype distribution; however, a higher proportion of non-B subtypes were found in the RS group (11.5% *vs *7.0%). Roughly 75% (14 out of 19) of recombinant samples were between B and F subtypes and none of them showed the same breakpoint of recombination in the *pol *region, or subtype determination between *pol *and *env *regions, confirming the high frequency of URFs BF, as usually described in our region [23].

An HIV-1 A_PR_UK_RT_A_ENV _mosaic genome was detected among the LTS. Based on the bootscanning analysis (data not shown), this viral genome was composed by a fragment of subtype A in the protease portion, one fragment of around 400 bp classified as unknown (U), located at the 5' side of the reverse transcriptase, followed by a subtype K fragment of 301 bp from position 2952 to 3253 (relative to HXB2 isolate). The gp120 C2-V3 region was assigned as subtype A.

## Discussion

Data generated from multiples studies conducted under the scope of the HIV/AIDS National Surveillance System, clearly indicate that the epidemic in Brazil persists as a concentrated one [3,4]. Like other countries in the region, our epidemic is driven mostly by Men who have sex with men [3,4]. The 4.8% overall prevalence found in our study conducted on three VCTs located in the outskirts of Rio de Janeiro State, are in accordance with data from studies conducted among clients seeking HIV testing in VCT services. These studies found a higher prevalence among these clients than in the general population in which rates are around 0.6%. [24-27]. Our data showing a prevalence of HIV infection 6 time higher among MSM when compared to heterosexuals participating in the same study, corroborates with a variety of studies which results showed high rated of HIV infection among this population (9.2% -32.2%). In a report of the Population Council, published recently, one study using the respondent-driven sampling (RDS) method points to a high prevalence (7%; 95%CI 5-11%) among MSM in the city of Campinas, state of São Paulo. Of special concern are the high prevalence found between very young MSM (aged 14-19) among whom a prevalence of 4% (95%CI 1-9%) was found, suggesting the epidemic in this population is far to be curbed [28]. Finally, all these data are in accordance with the AIDS national report system that register a proportion 11 times higher of MSM AIDS cases reported when compared to cases reported among heterosexual men [29], reinforcing the conclusion that MSM remain disproportionately affected by the epidemic in Brazil.

The approach adopted by our study has been found to be highly sensitive (82% [95%CI 77-86%]) and specific (89% [95%CI 85-92%]) [30], and has been used to estimate HIV national incidence in the USA [31], where viral diversity is similar to the scenario made evident for the regions assessed by the present study, where most of the infections are attributed to subtype B.

Different factors have been described as associated with BED-CEIA misclassification, which can lead to overestimated HIV incidence, including: HIV-infected individuals under antiretroviral therapy, patients with advanced immunodeficiency, drug naïve individuals with persistent low to undetectable viral loads, and different HIV subtypes [11,32,33]. In order to overcome these putative caveats we applied 2 correction factors (CF1 and CF2) to improve the incidence estimate rates in our study. Indeed, rates obtained after correction with CF1 and CF2 were close to each other and lower than the one obtained with the conventional method. Although lower estimates were obtained after the use of the correction algorithms, the proportions relative to the different categories remained approximately the same. This speaks in favor of the internal consistency of our findings, irrespectively of the inclusion or not of the correction factors. Recently, a series of publications discuss the pertinence and adequacy of such corrections [34-38] and, as to the best of our knowledge, this subject is still far from a consensus.

In our study, women found to be HIV-infected were almost invariably infected in consequence of unprotected sex with HIV infected male partners. This is a common pattern in contexts where blood transfusions are safe and HIV drug use is uncommon, like in Rio de Janeiro. This city metropolitan area has been relatively spared by the injecting drug users-driven boom of new HIV infections secondary to the shared use of injection paraphernalia, even in its national peak (in the late 1980s/early 1990s), and has declined since then. The number of female IV drug users has been particularly low [39,40]. Since early 90 s the standard of quality of the Brazilian blood banks is comparable to the best international ones [41]. As a result, new AIDS cases secondary to blood transfusions has been negligible in the last decades, specially in urban areas such as the one where our study was conducted (data available from Monitoraids and SINAN-AIDS at: http://sistemas.aids.gov.br/monitoraids/?lang=en-US&pagina=&st_lPanel=&indexMenu= -1).

No significant changes in the HIV-1 subtype distribution could be discerned among the RS compared to LTS. Subtype B was predominant, followed by subtype F and BF recombinant viruses, as previously described for the Brazilian Southeast region [42-44]. A different scenario may be observed for heterosexual men *vs*. women, when we compare HIV-1 infections with recombinant virus (almost 1:7 *vs*. 1:15, p = 0.19). One case of AUK recombinant infection was detected for the first time in Brazil.

## Conclusions

To the best of our knowledge, this is the most comprehensive assessment of HIV-1 prevalence, estimated incidence and subtype profile in the population seeking testing in the Rio de Janeiro metropolitan area and may contribute to better discern recent trends of the local epidemic. Moreover, our data suggests that prevention for MSM remains a challenge and efforts aiming to curb the epidemic in this population should be prioritized. The approach used for this study also permitted the identification of new HIV-1 isolate, as the AUK recombinant genome found for the first time in Rio de Janeiro, Brazil. Unfortunately, no further contact could be made with this individual in order to track the origin and the potential dissemination of this recombinant virus in the country.

Due to the fact our data were obtained from a population who actively sought HIV testing, findings cannot be generalized to the general population or to the sampling frame defined by men who have sex with men living in the metropolitan area of Rio de Janeiro and should be viewed with the necessary caution. Notwithstanding, the present study is the most comprehensive assessment of the population seeking testing in the Rio de Janeiro metropolitan area carried so far and may inform renewed policies aiming to prevent and treat people living with HIV/AIDS in Rio de Janeiro.

## Competing interests

The authors declare that they have no competing interests.

## Authors' contributions

CAVC, BG, VGV, FIB, JHP and MGM participated in the conception and design of the study; analysis and interpretation of data, drafting the paper and/or substantially revising it. All authors read and approved the final manuscript.

## Pre-publication history

The pre-publication history for this paper can be accessed here:

http://www.biomedcentral.com/1471-2334/10/224/prepub
